# A Customized Social Network Platform (Kids Helpline Circles) for Delivering Group Counseling to Young People Experiencing Family Discord That Impacts Their Well-Being: Exploratory Study

**DOI:** 10.2196/16176

**Published:** 2019-12-20

**Authors:** Andrew Campbell, Brad Ridout, Krestina Amon, Pablo Navarro, Brian Collyer, John Dalgleish

**Affiliations:** 1 Cyberpsychology Research Group Faculty of Medicine & Health The University of Sydney Camperdown Australia; 2 Kids Helpline Yourtown Brisbane Australia

**Keywords:** social media, social networking, online counseling, family discord, well-being

## Abstract

**Background:**

It has often been reported that young people are at high risk of mental health concerns, more so than at any other time in development over their life span. The situational factors that young people report as impacting their well-being are not addressed as often: specifically, family discord. Kids Helpline, a national service in Australia that provides free counseling online and by telephone to young people in distress, report that family discord and well-being issues are one of the major concerns reported by clients. In order to meet the preferences that young people seek when accessing counseling support, Kids Helpline has designed and trialed a custom-built social network platform for group counseling of young people experiencing family discord that impacts their well-being.

**Objective:**

In this exploratory study, we communicate the findings of Phase 1 of an innovative study in user and online counselor experience. This will lead to an iterative design for a world-first, purpose-built social network that will do the following: (1) increase reach and quality of service by utilizing a digital tool of preference for youth to receive peer-to-peer and counselor-to-peer support in a safe online environment and (2) provide the evidence base to document the best practice for online group counseling in a social network environment.

**Methods:**

The study utilized a participatory action research design. Young people aged 13-25 years (N=105) with mild-to-moderate depression or anxiety (not high risk) who contacted Kids Helpline were asked if they would like to trial the social networking site (SNS) for peer-to-peer and counselor-to-peer group support. Subjects were grouped into age cohorts of no more than one year above or below their reported age and assigned to groups of no more than 36 participants, in order to create a community of familiarity around age and problems experienced. Each group entered into an 8-week group counseling support program guided by counselors making regular posts and providing topic-specific content for psychoeducation and discussion. Counselors provided a weekly log of events to researchers; at 2-week intervals, subjects provided qualitative and quantitative feedback through open-ended questions and specific psychometric measures.

**Results:**

Qualitative results provided evidence of user support and benefits of the online group counseling environment. Counselors also reported benefits of the modality of therapy delivery. Psychometric scales did not report significance in changes of mood or affect. Counselors and users suggested improvements to the platform to increase user engagement.

**Conclusions:**

Phase 1 provided proof of concept for this mode of online counseling delivery. Users and counselors saw value in the model and innovation of the service. Phase 2 will address platform issues with changes to a new social network platform. Phase 2 will focus more broadly on mental health concerns raised by users and permit inclusion of a clinical population of young people experiencing depression and anxiety.

**Trial Registration:**

Australian New Zealand Clinical Trials Registry (ANZCTR) ACTRN12616000518460; https://www.anzctr.org.au/Trial/Registration/TrialReview.aspx?id=370381

## Introduction

The demand for Australian online counseling services by young people experiencing family discord and the impact on emotional well-being are substantial and unabating; 8.18% (12,052) and 9.04% (13,322) of the 147,424 contacts recorded by *yourtown* Kids Helpline in 2018 were related to family relationship problems and emotional wellness, respectively [1]. Family discord is defined as disharmony among family members, which may or may not include the child; this can include persistent arguments, controlling behaviors, intimidations, and threats [2]. Improved service reach using online platforms, such as group counseling via a secure social network, is a cost-effective approach to solving consumer demand owing to its accessibility to tech-savvy young people and it gives them the ability to respond en masse. However, challenges in ensuring appropriate user engagement, delivering high-quality evidence-based counseling, and maintaining the confidentiality and safety of clients are key to determining the efficacy of an online mental health service.

It is established that the most popular online resource young people are attracted to is social networking [3-5] and, yet, such platforms are currently not used at scale by mental health services to support young peoples’ mental health. This may be largely due to the problem with a lack of evidence-based implementation of social networking as a means of e-mental health service provision. Specifically, they are fraught with safety and privacy issues (ie, maintaining user anonymity as well as allowing counselors to monitor and intervene where necessary with *at-risk* clients). Of major note, globally, no research has been conducted in order to establish evidence-based policy and practice guidelines on how to group-counsel young people via a social network. Given this, online mental health support has typically been provided via chat, Web forum, or email-based counseling in a one-to-one, typically peer-to-peer, format by various mental health services. However, demand by young people for a secure and mobile phone-accessible mental health social networking service, where they can connect to counselors as well as peers experiencing similar issues, is growing given their online communication preferences [4,6]. In this study, we will use a custom-built social networking site (SNS) to address consumer demand as well as user preference for engaging with those with lived experience of family discord. While retaining the option to access counseling experts, this research aims to establish the viability and usability of Kids Helpline Circles (KHL Circles) as an innovative, purpose-built SNS delivered by *yourtown* Kids Helpline for Australian youth.

Kids Helpline is the leading national service in telephone and online counseling in Australia, given that it is the only 24/7 counselor-monitored service (ie, not a peer-to-peer counseling service). Consumers who receive help via the service often wish to revisit in order to receive ongoing counseling support, not just one-time counseling or further referral to other services. Garcia [7] reported that there were more than 70,000 attempts to contact Kids Helpline in the first half of 2019 that were not answered due to the service not having enough online and telephone counselors to meet the increasing demand. To provide more flexibility in meeting demand, Kids Helpline is now focused on providing a professionally facilitated online community for long-term support of clients between the ages of 13 and 25 years, to provide continuous connection and ongoing counseling support to prevent relapse. However, the concept of a purpose-built, secure, private, and counselor-controlled e-mental health-focused SNS needs to be piloted in order to ensure its efficacy before launching it as a new support service for Australian young people.

This participatory action research (PAR)-designed exploratory study [8] sought to develop the evidence base to validate the proof of concept for KHL Circles: a purpose-built, private, and secure SNS, designed to provide 24/7 group counseling to young people in Australia experiencing family discord. This study draws on evidence that young people already seek out others via their private social network choices (eg, Facebook and Instagram) for peer support, in order to feel they are not alone with their problems [4]. However, there are serious concerns that seeking help from peers online who are strangers and nonexperts may expose young people to inaccurate or misleading information and hostile or derogatory comments, which may have a negative impact on their mental health [9]. KHL Circles seeks to address these concerns by providing clients with evidence-based mental health information and support delivered via group counseling. If needed, counselors can direct young people to community services to access legal, financial, disability, or employment advice. KHL Circles also facilitates peer support by connecting clients with other clients of a comparative age who are experiencing similar issues to share stories and support each other; it is facilitated and monitored by professionally trained and accredited counselors who are part of their closed, small-group, social network. This supportive, moderated, online approach has been identified as theoretically optimal in several recent studies that have proposed SNS as an adjunct to online mental health interventions [6,10,11]. However, no research has yet determined a working model for utilizing a private and secure, purpose-built, e-mental health social network for ongoing group counseling to support young people experiencing family discord and mental well-being concerns.

## Methods

### Participants

A total of 105 participants were recruited from the Kids Helpline telephone and Web-chat counseling service, as well as from their website and social media posts. The participants initially contacted the service to seek help for their concerns and upon counselor interview and assistance for their immediate concerns, they were introduced to the option to join KHL Circles as volunteer participants. The inclusion criteria were as follows: (1) aged 13-25 years and of any gender identity, (2) newly contacting or previously engaged (ie, returning client) with Kids Helpline via one-on-one phone and/or Web counseling, (3) seeking support specifically for issues related to family discord and emotional well-being (eg, at-home psychological abuse, distress, or communication problems with family members), and (4) able to speak English (ie, required under ethical approval for the study, as no translator for other languages could be provided within the counselor-mediated social network). The mean age of participants was 16.2 years (SD 2.9) and the majority were female (86/105, 81.9%). The age, gender, and location breakdowns of participants are presented in Table 1. A total of 81.9% (86/105) of the sample spoke only English. Other languages also spoken included Mandarin, Cantonese, Dutch, Bosnian, Telugu, Punjabi, Bisaya, Korean, and Japanese.

**Table 1:** Participant demographics and response count.

Demographic		Participants (N=105), n (%)
**Age (years)**		
	13-15	50 (47.6)
	16-18	36 (34.3)
	19-21	10 (9.5)
	22-24	8 (7.6)
	25	1 (1.0)
**Gender**		
	Female	86 (81.9)
	Male	10 (9.5)
	Trans or gender diverse	6 (5.7)
	Other	1 (1.0)
	Missing	2 (1.9)
**Location**		
	New South Wales	32 (30.5)
	Victoria	22 (21.0)
	Queensland	22 (21.0)
	South Australia	4 (3.8)
	Tasmania	1 (1.0)
	Western Australia	1 (1.0)
	Australian Capital Territory	3 (2.9)
	Missing	15 (14.3)

### Design and Procedures

This exploratory study used a single-group, PAR, mixed-method design [8] to assess the acceptability, safety, user experience, and potential mental health benefits of KHL Circles. There were six *Circles* (ie, Groups) conducted over a 12-month period, from May 2017 to May 2018. There were 9 participants in Groups 1 and 2 (conducted concurrently), 8 participants in Group 3, and 13 participants in Group 4 (conducted concurrently with Group 3). After the first four groups were completed, the number of participants per group was increased to 32 for Group 5 and 34 for Group 6 in response to qualitative feedback from participants and to increase engagement and activity within each Circle.

Volunteer participants who contacted a Kids Helpline phone or Web counselor received immediate one-to-one counseling (ie, standard care model for the service). While in one-to-one counseling, they were asked if they would be interested in joining the study trialing the peer-support social network, KHL Circles. Those who indicated they wanted to join the trial were informed that they may not be able to join one of the Circles in the social network immediately and, if this was the case, they would be asked to wait for the next group to begin. Potential volunteer participants were screened for severe mental health problems (ie, high-level depression and anxiety) or risk of self-harm behaviors before being permitted into the online group-counseling environment. Screening was conducted through counselor interview. Those who were deemed high risk were provided with one-to-one counseling via Kids Helpline’s regular telephone or Web counseling services. Once suitable participants were assigned to a KHL Circle, they completed an entrance survey containing psychometric tests to measure baseline depression, anxiety, self-esteem, and perceived social support.

All volunteer participant members of KHL Circles were asked to use pseudonyms in order to protect their identities from each other. The counselor facilitating the group was the only group member aware of their true identities. Participants were instructed not to reveal any identifying information during the trial, including any identifying photos or images. Those recruited were asked to give electronic consent if they were 16 years of age or older, or consent and optional assent from a parent or guardian if 15 years of age or under. Participants were also asked to read the Kids Helpline policy and agree that during their time in KHL Circles, if they choose to start their own social network support group on a non-Kids Helpline service (eg, Facebook), that Kids Helpline would not be liable for the safety and running of those groups.

Participants completed a baseline survey in the week prior to joining their Circle containing psychometric tests measuring levels of depression, anxiety, self-esteem, and perceived peer support. Participants were also asked to complete a *check-in* survey at the end of weeks 2, 4, and 6, and a final survey at the conclusion of the study (ie, end of week 8); all surveys contained the same four psychometric tests, along with open-response questions on their experiences of KHL Circles. Counselors provided weekly reports on their perceptions of group engagement and any group-counseling concerns or functional issues regarding the online platform. All participants were informed prior to joining KHL Circles that their Circle would be closed at the end of the 8-week cycle, after which each member may revert to one-to-one counseling using Kids Helpline telephone and Web counseling services.

The research protocol was approved by The University of Sydney Human Research Ethics Committee (HREC) (Project #2016/132) and registered with the Australian New Zealand Clinical Trials Registry (ANZCTR) (ACTRN12616000518460).

### Kids Helpline Circles Platform

KHL Circles was developed by a team of researchers, psychologists, and programmers following consultation and beta testing with young Kids Helpline clients and using PAR design principles [8]. The platform was developed using the open-source social networking software Elgg [12], which was customized by a team of Web developers and graphic designers to meet the requirements of the service. The platform was available to participants via any Internet-enabled computer or mobile device. The mobile version of the site was adjusted to fit the size of the screen being used by the participant but included all the same components as the desktop version. Participants could log in to KHL Circles anytime throughout the trial. The platform was run on Kids Helpline’s own private servers and monitored by Kids Helpline counselors 7 days a week. Clients were also asked to report any risk they foresaw or major conflict within the group by contacting Kids Helpline via telephone or Web counseling services.

**Figure 1:**
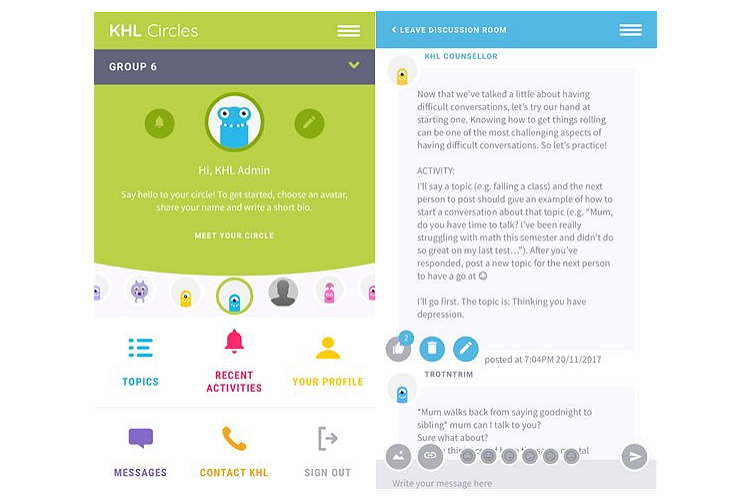
Kids Helpline Circles (KHL Circles) mobile phone interface design.

As part of the baseline survey, participants put forward their pseudonym, which was reviewed by the site administrator to ensure that it did not reveal their identity. In the days leading up to week 1, the site administrator assigned participants to their Circles and emailed them unique log-in details. Participants were asked to log in to the site prior to the week 1 commencement date to complete their profiles (ie, gender, hobbies, and likes) and to choose a profile picture from a suite of *alien* images (see Figure 1 for images of some of the alien characters for user choice). On the first day of week 1, a KHL counselor posted a welcome message explaining to participants how the Circle would be run and asked them to test out the posting features of the site (ie, posting text, pictures, and videos, and *liking* or commenting on posts of other users, similar to well-known Facebook functions; see Figure 1). Participants were also provided with some ground rules (eg, respecting others, no offensive material, and keeping their identity private) and were asked to expand on this list with their own expectations of their Circles.

The main menu bar of the site presented six tools:

1. Topics: the default view, which showed a list of the all discussion posts and threads posted to date, with the most recent at the top of the page.
2. Recent activities: allowed participants to quickly access topics they had contributed to.
3. Your profile: participants could update their profile information here.
4. Messages: participants could send and receive a private message to or from a Kids Helpline counselor but not to or from other participants.
5. Contact KHL: a link to the Kids Helpline phone and Web-chat counseling service.
6. Sign out.

For each Circle, Kids Helpline Counselors posted three new *topics* each week—every Monday, Wednesday, and Friday—consisting of age-appropriate psychoeducational material about family discord, including conversational text, images, and videos; topics also consisted of a reflection and discussion activity to encourage engagement and interaction between participants on ways of addressing various issues within this subject. Each week focused on one of eight modules: (1) Introduction; (2) Family relationships; (3) Emotions; (4) Mental health and resilience; (5) Help-seeking and social support; (6) Family communication and negotiation skills; (7) Conflict resolution, self-care, and relapse prevention; and (8) Summary and close. Participants could also start their own *topics* or contribute to an ongoing *Song of the Day* topic.

### Safety Protocols

The safety protocol of KHL Circles is based upon the existing safety protocols of the Kids Helpline phone and Web counseling service, which includes policies for mandatory reporting. Monitoring of KHL Circles by Kids Helpline counselors was done 24/7 and was combined with having access to contact details via particpants’ Kids Helpline files; this allowed any potential risks to the safety or mental health of participants to be addressed proactively by contacting the participant directly via email or via the private messaging function of KHL Circles, encouraging them to contact the Kids Helpline phone counseling service.

KHL Circles was hosted on Kids Helpline’s private server, using a URL secured with HTTP over Secure Socket Layer (SSL); HTTP Secure (HTTPS) conformed to industry best practice as defined by the Open Web Application Security Project [13]. Privacy and online safety were managed in accordance with recommendations by the eSafety Commissioner of the Australian Government [14], with participants required to accept the terms of use of KHL Circles, which included clauses regarding their privacy, online bullying or harassment, and the use of offensive comments. Participants were informed that failure to comply with guidelines may result in temporary or permanent removal from the service. In accordance with international standards for the legal age of having a social media account, only those 13 years of age and over were permitted into the study. Moreover, in accordance with The University of Sydney HREC and the ANZCTR, those aged 13-15 years needed to provide personal assent, while those 16 years of age and over needed to provide personal consent. All participants under the age of 18 years were informed that they had the option to gain parental consent for the study.

### Measures

The following psychometric tests were included as part of each survey (ie, the baseline survey; *check-in* surveys at weeks 2, 4, and 6; and the final survey at week 8): (1) the Multidimensional Scale of Perceived Social Support [15], (2) the Centre for Epidemiological Studies—Depression Scale for Children [16], (3) the Revised Children’s Manifest Anxiety Scale [17], and (4) the Rosenberg Self-Esteem Scale [18].

The baseline survey asked participants what they hoped to gain from KHL Circles (open response). Each check-in survey and the final survey asked participants whether they felt that KHL Circles had helped them feel supported in coping with their problems (yes/no) and, if so, what made them want to return to communicate with their Circle (open response). The final survey also asked participants to indicate how helpful they found KHL Circles (4-point scale), if they would return to KHL Circles for any future issues (yes/no), and what sorts of issues they would be comfortable discussing in KHL Circles (from a list of nine options or specify *other*). Participants were also asked to indicate the most helpful and least helpful aspects of KHL Circles (from a list of four options for each or specify *other*) and what they considered to be the most important features of a social media peer-support site (from a list of eight options or specify *other*). Each survey concluded with the opportunity for participants to provide any other comments or feedback (open response).

Safety was assessed using the following a priori indicators: (1) any instances of adverse events, (2) any instances of breaching guidelines of use, and (3) qualitative feedback from participants.

## Results

### Participants

As recorded in the baseline survey, most participants were female (86/105, 81.9%) and aged between 13 and 15 years (43/105, 41.0%). A total of 105 participants responded to the baseline survey; however, over the course of the study, the number of participants who completed the *check-in* surveys and the final survey (week 8) reduced significantly (see Table 2).

**Table 2:** Participant demographics and response count.

Questionnaire	Participants (N=105), n (%)
Baseline survey	105 (100)
Week 2 survey	48 (45.7)
Week 4 survey	12 (11.4)
Week 6 survey	13 (12.4)
Final survey (week 8)	8 (7.6)

### Qualitative Data Analysis

A simple content analysis of responses to the qualitative questions was conducted. Each response was read and assigned an open code that summarized the key idea in their initial response [19,20]. For example, “to see if some other people are in my situation so I don’t feel like my problems only apply to me and my family” was assigned an open code of *Relate with others*. Similar codes were grouped together under axial codes, which were given a descriptive heading. For example, *Relate with others* was grouped with *Not to feel alone* under *Connect with others*. Similar axial codes were then grouped together under main categories and provided with a descriptive title. For example, *Connect with others* was grouped with *Receive support* under the category *Engage with others for support*. KA reviewed all coding and BR provided a second review: each was a research associate covered under HREC approval for the study analysis. Disagreements were resolved by consensus [21].

As part of the baseline survey, participants were asked, “What do you hope to gain from joining Kids Help Line Circles?” Content analysis of the 105 participant responses to this question produced six categories: (1) *Engage with others for support* (76/105, 72.4%; eg, “I hope to gain support through meeting people going through similar experiences and not feeling as alone”); (2) *Receive information* (26/105, 24.8%; eg, “An insight into how others deal with similar circumstances”); (3) *Positive self-outcome* (22/105, 21.0%; eg, “Something that can make me feel happy and worthy of myself”); (4) *Provide support* (11/105, 10.5%; eg, “I hope to gain a better mindset and help others that are in trouble”); (5) *Miscellaneous* (3/105, 2.9%; eg, “I hope that it’s worth joining”); and (6) *Unsure* (8/105, 7.6%).

As part of the *check-in* surveys, participants were asked, “Do you feel that being a member of the KHL Social Network 'Circles' has helped you feel supported in coping with your problems?” to which 41 out of 68 (60%) responses were *yes*. Participants were further asked, “If being part of Kids Helpline Circles has helped you, what makes you want to return to talk to people when you feel like it?” Content analysis of 22 participants’ open responses revealed four categories: (1) *Sense of community* (15/22, 68%); eg, “I feel like others understand what I am going through, and that I am not alone in my feelings and struggles”); (2) *Safe environment* (6/22, 27%; eg, “Knowing that it’s a safe environment to help others and to get help”); (3) *Helpful environment* (5/22, 23%); eg, “All the people I have talked to is [sic] really nice and I feel like we all really make an effort to help and support each other in any way we can”); and (4) *Miscellaneous* (2/22, 2%; eg, “I don't use it much but I will try to more”).

The final survey also asked participants, “Do you feel that being a member of the KHL Social Network 'Circles' has helped you feel supported in coping with your problems?” to which there were 5 responses, 3 (60%) of which were *yes*. Only one participant provided a response to the follow-up open-response question “If being part of Kids Helpline Circles has helped you, what makes you want to return to talk to people when you feel like it?” so qualitative analysis was not possible.

### User Experience

As part of the final survey, participants were asked questions about their experiences using KHL Circles. Of the 8 participants who responded to the final survey, 5 (63%) provided responses to the user experience questions. The topics participants felt most comfortable discussing were peer relationships (3/5, 60%), family relationships (3/5, 60%), depression (3,/5 60%), and anxiety (3/5, 60%). Other responses included well-being, motivation, sexuality, romantic relationships, gender and identity, and image. When asked, “What did you find the most helpful aspect of KHL Circles?” responses included “connecting with people who understood my concerns” and “learning a lot about the topic discussed.” The only unhelpful aspect identified was “focusing on just the topic” (ie, family discord). When asked about what they thought the most important features of a social media peer-support service were, the most common response was *emojis* (4/5, 80%), followed by *games* (3/5, 60%), *anonymity* (3/5, 60%), *mobile phone app integration* (3/5, 60%) and *easy navigation* (3/5, 60%).

### Weekly Summary Reports by Counselor Facilitators

In addition to the questionnaires completed by participants, the counselor facilitators submitted weekly summaries to report on participant activity and user experiences. In each Circle, activity was highest in week 1, with 207 posts or comments and 532 *likes* in total across all groups. Activity generally started to drop off by week 2 (170 posts or comments and 326 *likes*), with the biggest drop during week 3 (73 posts or comments and 128 *likes*). Engagement typically continued to reduce over the remaining weeks, reaching a low of 11 posts or comments and 69 *likes* in week 8. Counselors reported a pattern where many participants were logging on and participating earlier in the week but not returning during the remainder of the week.

The most frequent comments made about the experience using the platform were about the navigation challenges. These included clunky scrolling mechanics, inability to resize images, manual linking of images and videos, and inability to archive posts for better flow. Frequent mention was also made about the need to include an automated time stamp to orient users about when posts were made.

Simple technical issues raised by participants involved issues uploading images and videos and the changing of passwords, which were guided or corrected by the counselors directly. Issues that needed immediate attention included counselors not being able to see groups due to log-in or log-out errors and an inability to directly message participants via Elgg, unless participants had directly messaged them first. One participant also suggested to counselors that mobile app notifications were needed, which was a function that was unfortunately not available within the Elgg platform.

### Psychometric Tests Analysis

An intention of this exploratory study was to conduct repeated-measures quantitative analyses of the psychometric tests administered to participants; however, due to the drop-off in response rates between the baseline survey (105/105, 100%) and final survey (8/105, 7.6%), data quality was too low to conduct meaningful analysis.

## Discussion

### Principal Findings

This exploratory, mixed-methods, PAR study aimed to assess the user experience and potential benefits of a purpose-built social networking platform, KHL Circles, for online group counseling of young people experiencing family discord. Results showed that the main benefit participants hoped to gain from KHL Circles prior to joining was engaging with others with similar lived experiences. This was the most common theme identified in relation to engaging with others for support. Other themes identified included gaining new information and positive self-outcomes. While low response numbers precluded any significant findings from the psychometric measures used in the study, the majority of participants reported that the overall experience of being a member of KHL Circles helped them in being supported in coping with their problems with family discord. Of those who reported that the service did not help them, it was found that comorbid problems not specific to family discord may have been a factor (eg, school bullying, romantic relationship problems, and specific mental health concerns). While the response rate to the final survey was very low, the majority of those who did remain engaged through the full 8 weeks reported they would continue to use the purpose-built social network if it was made available, which qualitative responses indicated was largely due to obtaining a sense of community about a specific issue they all shared.

As reported by the counselor facilitators of KHL Circles, the least helpful aspect of KHL Circles was reported to be the Elgg platform itself, given that it was not easy to navigate or find threaded responses for counselor engagement with each group. Many users reported that the platform would have been more engaging if it was like existing, popular social networks that they were familiar with (eg, Facebook) and provided a better quality of standard tools (eg, emojis, games, and better mobile phone operability).

### Limitations

While providing proof of concept for the KHL Circle model, results from this study are limited by low retention rates across the 8-week cycle of each group. User experience feedback should, therefore, be interpreted with caution as it only reflects the experiences of participants who completed the entire 8-week cycle. Completion rates of all psychometric surveys were poor beyond initial baseline collection (see Table 2), so no inferences could be drawn regarding any impact on mental health and emotional well-being. Low engagement with these surveys is not surprising given the age group studied and their primary motivation to be part of the study (ie, to connect with others the same age with lived experience of family discord). The length and clinical focus of the surveys—those not focused on family discord issues—may also have led to refusal to complete the surveys due to self-perceived lack of relevance, disinterest, or disengagement from the study. Phase 2 of KHL Circles will seek to significantly reduce the number and length of mental health surveys provided, as well as to make changes to the platform to increase engagement and activity within the groups in order to encourage higher retention across the 8-week program.

The recruitment for this study was restricted to those with mild-to-moderate levels of depression, anxiety, or stress, in order to abide by the strict ethics protocol provided for this exploratory research. As such, through careful ethical consideration of online safety of minors, this study may have inadvertently denied access to those who are in significant need of such an innovation to aid their distress around family discord and provide relatable online community support and expert counselor facilitation. Phase 2 will seek the inclusion of all young people who wish to access the KHL Circles service, given that Kids Helpline’s services already attract a high level of distressed young people that would not fit the category of mild-to-moderate levels of depression and anxiety.

Of importance to note was the very high rate of female participants in this study. While not unusual to see more females than males engage in seeking help [22]—historically, yourtown Kids Helplines’ client data over two decades supports this trend—ways in which to attract males to online services needs to be further explored. This could include the introduction of more *project*-oriented group counseling set around a focus activity such as gaming, for example [23].

From a technical perspective, the Elgg platform was reported by users and counselors to be too rigid to use in comparison to popular platforms such as Facebook. Given this, at the conclusion of the Phase 1 study, the researchers sourced a new platform called HumHub [24], which will undergo customization by Kids Helpline for Phase 2. It includes high-level server security features and functions requested by users (ie, emojis, better integration with linked images and videos, and notifications), with an interface like publicly available SNS’s such as Facebook. Of notable importance for this research progressing into Phase 2 will be the continuous monitoring of user experience of the social network service platform in order to incorporate iterative design changes to meet the needs of both the end user (ie, client) and the counselor facilitator.

### Conclusions

Social network uptake and sustained use by young people, not only for leisure but for community support or nonexpert counseling and advice-seeking, has been documented over many years [4,5,25-27]. The risk of utilizing nonexpert, nonsecure, social network support groups via such platforms as Facebook is problematic at best and dangerous at worst for the well-being and safety of young people [25,28]. Kids Helpline’s innovation in developing a custom-built, social network platform focused on the two most common topics young people contact their services around: family discord and mental well-being. Phase 1 is the first phase of a series of PAR studies to refine such a resource to address typical youth issues. Thus, the findings from this study support proof of concept and user interest for its evolution as a service delivery model. The potential significance of the proposed research is the provision of greater access to online support for clients of Kids Helpline and other online mental health services globally; lack of access is highly problematic, given that young people often do not know how to access mental health services as first-time users, find the services on offer costly and invasive to their needs, or simply do not trust what is available, whether it is online or offline [3-5,9,11,22,25,27,29].

As determined by Ridout and Campbell’s [6] systematic review, this research is the first of its kind in determining an innovative online model for cost-effective provision of short-to-long-term psychosocial support, with potential for ongoing group counseling support of Australian youth with mental illness. The applied research approach between academic experts, clinical expertise, and a not-for-profit group—yourtown Kids Helpline—demonstrates clearly the recommendations of utilizing a partnership model in innovating and developing mental health services for hard-to-reach populations (eg, youth) [29]. This research has moved on to Phase 2, where data collection began in 2018 and will continue through 2020, during which the implementation of the revised platform will be evaluated with the inclusion of a larger and more diverse user group, inclusive of young people experiencing greater-than-moderate levels of distress.

## Abbreviations

ANZCTR: Australian New Zealand Clinical Trials Registry
FGX: Future Generation Investment Fund
HREC: Human Research Ethics Committee
HTTPS: HTTP Secure
KHL Circles: Kids Helpline Circles
PAR: participatory action research
SNS: social networking site
SSL: Secure Socket Layer
